# Preparation of the Mouth for Artificial Dentures

**Published:** 1880-01

**Authors:** John Lauder

**Affiliations:** Montreal.


### ARTICLE V.

### *Preparation of the Mouth for Artificial Dentures.*

BY JOHN LAUDER, L. D. S., D. D. S., MONTREAL.

It cannot be denied but that a large proportion of artificial dentures are not worn with perfect comfort. A good many are merely tolerated, and a fair proportion not used at all, but thrown aside as uncomfortable.

I propose in this paper to examine some of the causes which lead to misfits, and some of the precautions to be taken preparatory to their insertion.

#### 1. PARTIAL SETS

Demand more care and skill than full dentures. Sufficient attention is not paid to the complete absorption of the gums and alveolus, and the removal of salivary calculus in standing teeth. It is necessary to remove all tartar from the necks of the teeth; but to secure a particularly good fit, where the tartar has accumulated to such an extent as to intrude upon and inflame the gums, I would remove it *some days*

*before taking the impression;* because if the impression is taken at the same sitting, sufficient time is not given for the subsidence of inflammation and the change which is sure to follow, and which is quite sure when it occurs after the set is inserted to alter the nice adaptation of the plate at the margins of the gums. In fact, where the gums are swollen in any case about the necks of the teeth, either from simple inflammation, the discharge of purulent secretion, etc., a few drops of equal parts of Fleming's Aconite, and Liniment of Iodine should be painted on the gums before taking the impression.

Care must be taken not to leave in loose teeth which would destroy any good fit in course of time. It may at times be allowable and perhaps preferable to leave a healthy root. No rule but the judgment of the dentist can dictate. Of course any cavities in standing teeth should be filled and the patient instructed to use the most absolute cleanliness when wearing the set.

One very sound reason why a root should occasionally be left, is that after its removal the adjacent teeth change their position if the plate is not constantly worn; and if the plate should be left out for any length of time by reason of illness or otherwise, even the articulation of these teeth with their lower antagonists is in a measure destroyed. A root may be often an invaluable retainer; healthy roots are no more liable to become diseased than before the crown was destroyed—that is, if the pulp was dead. If left in they should be dressed down with the corundum wheel on the engine as for pivot teeth.

It is a custom in preparing for upper sets, where all the other teeth have to be removed, to sacrifice the cuspids in almost every case, even though they should be sound. Nature has implanted these teeth like the pillars of an arch to preserve the contour of the face. The length and the prominence of their roots show that they are specially adapted to preserve the shape of the face. I prefer to save them whenever possible, because they preserve this contour, and prevent the ugly falling of the face under the nostrils, where no artificial substitute ever can extend high enough to remedy the defect. Blocks of four can be obtained or carved to be fitted neatly in one section to the space between the cuspids. Of course it is supposed that these teeth are really worth preservation, and are of good enough structure to justify their retention. I have very often found this to be the case, and I may here say, that a set skillfully made in this way is worth a larger fee than the ordinary full upper set.

FULL SETS.—UPPER OR LOWER.

As a general thing the roots should be entirely removed when a single full set is to be inserted on atmospheric pressure. There may be circumstances in old people where springs could be used, and the healthy roots left. In the older countries of Europe, roots seem to last better than here, and the pulp cavities are often opened, filled with gold, and may remain for many years; but a perfect fitting set cannot be thus made, as nature effects the ultimate dislodgment of the roots, and as they are ejected they cause the plate to loosen.

There may be special reasons, such as ill-health—when all healthy roots should be retained. Ordinary cases are treated in an ordinary way; but take mouths where there is a great protrusion and excess of the alveolus with a short lip. No matter how thoroughly the mouth heals, it is not probable that the absorption will be sufficient to allow of the insertion of *gum teeth* at any time. There may be cases where the alveolus has absorbed so little at the upper part *under the nostrils* that the lip does not recede at this point, and plain teeth may be used; but the larger proportion turn out quite the reverse, and while the bone changes but little at the lower front, a deep absorption results just where it is most noticed, and just where no substitute can be applied on account of the shortness of the. lip, which would expose any vulcanite placed over front teeth. To forestall this difficulty in such a case I would prefer, after the extraction of the teeth, to cut down the alveolus with alveolar or excising forceps, from the second bicuspid on both sides towards the front; dissecting the gum if necessary, and cutting both inner and outer plates as well as removing the transverse processes and adjacent sockets. The result must be that the absorption is greater, and the mouth a better shape eventually than if all the sockets, etc., were left to the slow and natural process of change.

Another alternative may be resorted to in some cases. If the roots of the ten teeth can be preserved in a healthy condition by excision and dressing down, and plugged; and then plain teeth adapted over these, with gum molar blocks or wings behind, the difficulty may be overcome.

The *den. sapentiæ* ought to be extracted invariably. They are no sort of use in any case, and ninety times out of a hundred if left, only involve the loss of suction and the renewal of the set at some future time.

The question of retaining roots has provoked a good deal of discussion in England. It is held there that by their removal you destroy the shape of the face, as well as the alveolar ridge. As I said in a previous part of this paper, there may be special cases where the retention of healthy roots is desirable and even preferable; but it seems a fact that roots do not remain as healthy in America, as in Europe, and that we cannot follow the practice here as indiscriminately; and even those who advocate in Europe their retention, admit in many cases that any artificial substitute can only be temporary, placed over roots; that the time must come when they will cause trouble, and that their extraction has at last to be resorted to. Yet there are more serious arguments against their retention. If patients have to wear artificial teeth at all, the earlier they become accustomed to the greater bulk of gum teeth and vulcanite, the better will the plate be worn. Besides a plate worn over roots has no lasting comfort. It is also always exposed to fracture over the roots as the roots elongate. Another objection may be offered, viz., that neuralgic pains in the head, nausea, and bad breath are frequently present when roots are retained. Obscure pains in the head may be often traced to this cause.

After extraction where roots have been ulcerated and pus has discharged, the patient should be instructed to rinse the mouth thoroughly several times a day with water as hot as it can be borne, and indeed the hot water after extraction, used frequently, is one of the most useful applications to reduce inflammation and tenderness.

After a couple of days of this treatment I would recommend an astringent wash of Pyrethrum Root.

Where the patient has been chloroformed, I recommend a Turkish bath, as tending to cleanse the system of its influence.—*Canada Journal Dental Science.*

---

